# Numerical simulation of oil spill dispersion and weathering in the Alboran Sea: A case study of its North-Eastern Coast

**DOI:** 10.1371/journal.pone.0358443

**Published:** 2026-09-25

**Authors:** Asmae Nouayti, Soufiane Oubdil, Sara Ajmani, Safae El Aammouri, Payâgda Misaël Tarpaga, Lhoussaine Kammou, Mounaim Halim El Jalil, Mohammed Al-zharanie, Fahd A. Nasre, Ashraf Ahmed Qurtame, Ali Ait Boughrous, Nadia Lahrach

**Affiliations:** 1 Ethnopharmacology and Pharmacognosy, Faculty of Sciences and Techniques Errachidia, Moulay Ismail University of Meknes, Errachidia, Morocco; 2 Doctoral Studies Center of Engineering Sciences and Techniques, Mohammadia School of Engineers (EMI), Mohammed V University of Rabat, Rabat, Morocco; 3 Civil Engineering and Environment Laboratory (LGCE), Materials Water and Environment Team, Higher School of Technology of Mohammed V University of Rabat, Rabat, Morocco; 4 Laboratory of Vegetal, Animal Productions and Agro-Industry, Faculty of Sciences, Ibn Tofail University, Kenitra, Morocco; 5 Biology Department, College of Science, Imam Mohammad Ibn Saud Islamic University (IMSIU), Riyadh, Saudi Arabia; 6 Bio-resources, Environment and Health, Faculty of Science and Technology of Errachidia, Moulay Ismail University of Meknes, Meknes, Morocco; Universidade de Aveiro, PORTUGAL

## Abstract

Accidental oil spills in the Alboran Sea represent a high-risk hazard because intense maritime traffic, semi-enclosed circulation, and sensitive Moroccan coastal ecosystems converge within a narrow coastal corridor. Operational-scale simulations for the Nador-Saïdia sector remain scarce, particularly for persistent heavy fuel oil and for the combined effects of mesoscale circulation, wind drift, Stokes drift, vertical mixing, and weathering. This study applies the OpenOil module of the OpenDrift Lagrangian framework to simulate a hypothetical release of 100 tons of heavy fuel oil (Generic Bunker C) offshore the Driouch coast (35.45° N, 2.95° W) over a seven-day (168 h) period, beginning on 02 July 2024 at 12:00 UTC. The model was forced with CMEMS Mediterranean physics fields, CMEMS Mediterranean wave data, and NOAA-GFS 10 m winds. The selected summer scenario represents a high-impact vulnerability case characterized by strong stratification, sea-breeze influence, peak beach-tourism exposure, and high ecological sensitivity of the Marchica Lagoon, Moulouya River estuary, and Cap des Trois Fourches. The simulations incorporated high-resolution meteorological and oceanographic forcing from CMEMS and NOAA-GFS. Results highlight the strong influence of mesoscale circulation patterns in the Alboran Sea, particularly the interaction between regional gyres, the Algerian Current, and local wind-driven drift. Lagrangian trajectories indicate a dominant east–southeastward transport, posing a direct threat to environmentally sensitive coastal areas. By the end of the simulation, 4,253 particles were stranded, corresponding to approximately 85.1 t of oil, while 747 particles remained active at sea, corresponding to approximately 14.9 t. Weathering was dominated by emulsification and viscosity increase, whereas evaporation remained limited to approximately 10–15% of the released mass and natural dispersion remained below 10%. The revised analysis indicates that high viscosity and density lower than seawater explain the long surface residence time of fresh Bunker C, while sedimentation risk becomes critical mainly in shallow, sediment-rich environments through oil-mineral aggregation. The study identifies priority coastal exposure pathways and provides management-relevant guidance for preparedness planning, including pre-positioning of response equipment adapted to viscous oils, rapid protection of lagoon inlets where feasible, and transboundary coordination between Morocco and Algeria.

## 1. Introduction

The Mediterranean Sea is a semi-enclosed basin linking the Atlantic Ocean to the Indian Ocean via the Suez Canal and represents one of the most intensively used maritime corridors worldwide. Although it accounts for only about 0.7% of the global ocean surface, it accommodates nearly 30% of global commercial maritime traffic and approximately 20% of worldwide seaborne hydrocarbon transport [1,2]. This exceptional concentration of maritime activity, combined with the confined geometry of the basin, substantially increases the vulnerability of the Mediterranean Sea to accidental oil spills resulting from ship collisions, groundings, or illegal discharge operations [3].

Integrated coastal zone management and the protection of marine ecosystems from anthropogenic pressures have therefore become critical challenges for maritime nations in the twenty-first century. Among these pressures, hydrocarbon pollution—whether chronic or accidental—poses a persistent threat to marine biodiversity, the blue economy, and public health. Owing to its strategic position at the interface between the Atlantic Ocean and the Mediterranean Sea, Morocco is particularly exposed to the intensification of maritime traffic, especially in the Alboran Sea, a key transit area for international commercial shipping.

Oil pollution is particularly problematic in the Mediterranean because hydrocarbons can be transported rapidly between national jurisdictions, while the residence time of contaminants may be prolonged by coastal retention, mesoscale recirculation, weak tidal flushing, and recurrent wind-wave events. Hydrocarbon pollution threatens marine biodiversity, coastal tourism, fisheries, aquaculture, and public health [4]. Drawing on insights from major pollution events, such as the 2019 oil spill along the Brazilian coast [5]. For southern Mediterranean countries, including Morocco, preparedness therefore requires predictive tools that can translate metocean forecasts into operational exposure maps within hours to days after a spill.

The north-eastern Moroccan coastline (NEMC) is characterized by exceptional marine and coastal natural resources, including ecologically sensitive habitats, spawning and nursery areas for numerous fish species, and coastal systems that play a vital role in shoreline protection as described by [6] in his recent study. This region hosts several sites of national and international conservation importance, notably the Cap des Trois Fourches, designated as a Site of Biological and Ecological Interest (SIBE), the Marchica Lagoon, one of the largest lagoonal ecosystems in the Moroccan Mediterranean and classified as a Ramsar site, and the Moulouya River estuary, also designated as a Ramsar site and recognized as one of the most significant wetlands in north-eastern Morocco [7,8].

Lagrangian particle-tracking models are widely used to simulate the drift and fate of oil slicks because they resolve point-source releases, strong concentration gradients, and the individual histories of oil elements under time-varying forcing [9,10]. In complex coastal environments, accurate simulations require the combined representation of ocean currents, wind drift, Stokes drift, turbulent diffusion, vertical mixing, wave entrainment, resurfacing, evaporation, emulsification, and shoreline stranding. These requirements are especially important for heavy fuel oils such as Bunker C, whose high viscosity reduces natural dispersion and complicates mechanical recovery.

In this context, the present study applies the OpenOil module integrated within the OpenDrift Lagrangian framework to simulate the transport, dispersion, and weathering of a hypothetical Generic Bunker C spill along the north-eastern Moroccan Mediterranean coast. The novelty of this work lies in the integration of operational meteorological and oceanographic forcing with a process-based analysis of oil transport pathways, weathering evolution, vertical mixing, and shoreline exposure within a coastal sector of high ecological and socio-economic importance.

The specific objectives of the study are to: (i) simulate the seven-day evolution of a hypothetical heavy-fuel-oil spill offshore the Driouch coast; (ii) quantify the temporal evolution of floating, stranded, dispersed, evaporated, and biodegraded oil fractions; (iii) investigate the respective roles of winds, ocean currents, waves, and Stokes drift in controlling horizontal and vertical oil transport; and (iv) identify the coastal sectors most exposed to potential hydrocarbon contamination in order to support preparedness and response planning.

## 2. Study Area

**Fig 1:**
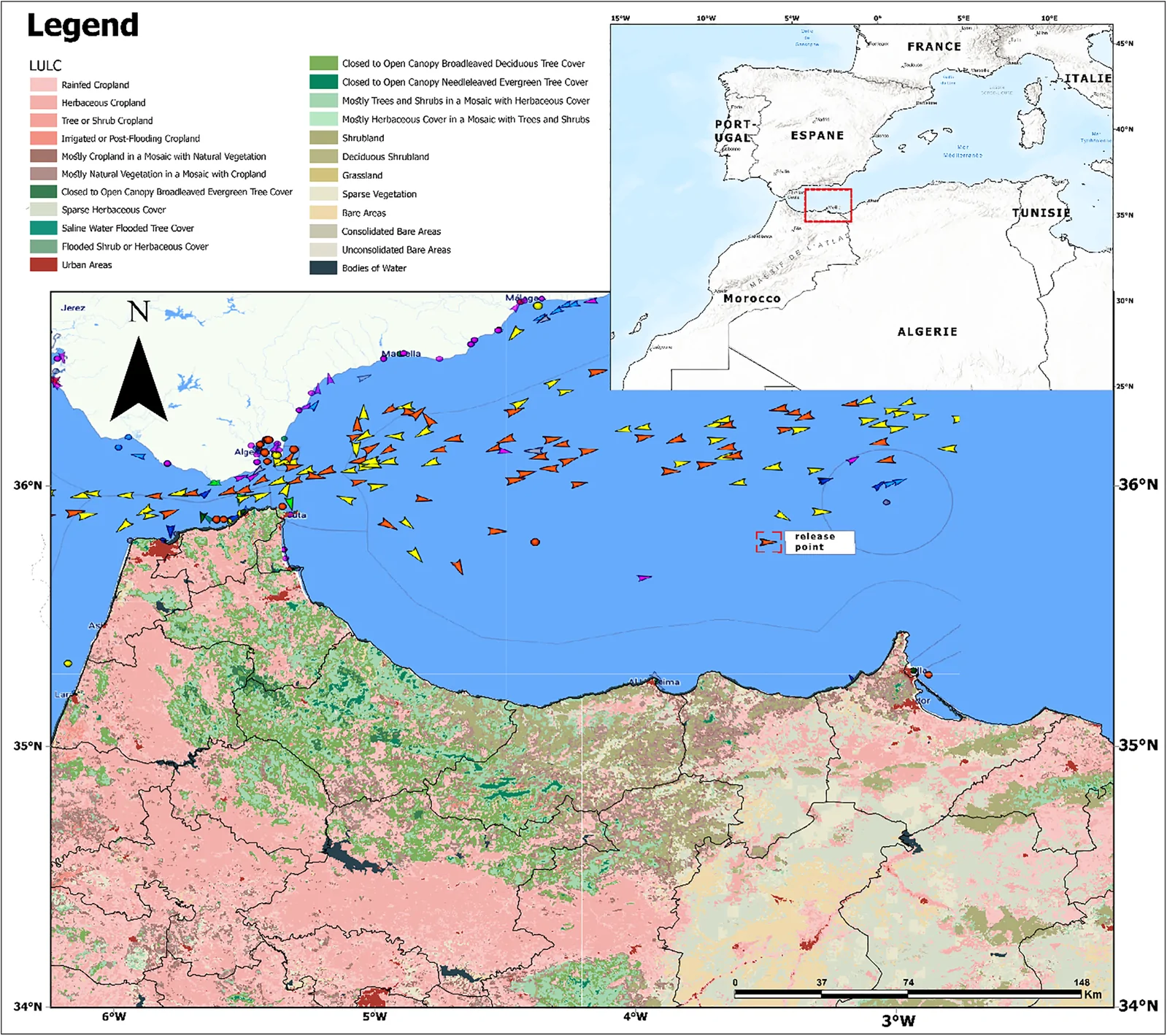
Study area and location of the simulated oil-spill release point along the north-eastern Mediterranean coast of Morocco. Base map data © OpenStreetMap contributors. Land-cover information was derived from publicly available land-cover datasets. Vessel traffic information was obtained from MarineTraffic (https://www.marinetraffic.com, accessed on 05 July 2025).

The study area is located along the NEMC, encompassing the provinces of Driouch, Nador, and Berkane (Fig 1). The release point for the numerical simulations is positioned offshore of the Driouch coastline at 35.45° N and 2.95° W, a sector exposed to traffic moving between the Strait of Gibraltar, western Mediterranean ports, and eastern Mediterranean/Suez routes. The coastline includes rocky headlands, sandy beaches, estuarine wetlands, and the Marchica Lagoon inlet system, creating a heterogeneous shoreline with variable retention and clean-up accessibility. This Mediterranean region exhibits a high degree of bathymetric and hydrodynamic complexity, exceeding that of the Moroccan Atlantic coast [11]. The regional circulation is dominated by the dynamics of the Alboran Sea, driven by the inflow of Atlantic surface waters that form two semi-permanent mesoscale structures: the Western Alboran Gyre (WAG) and the Eastern Alboran Gyre (EAG) [12].

**Fig 2:**
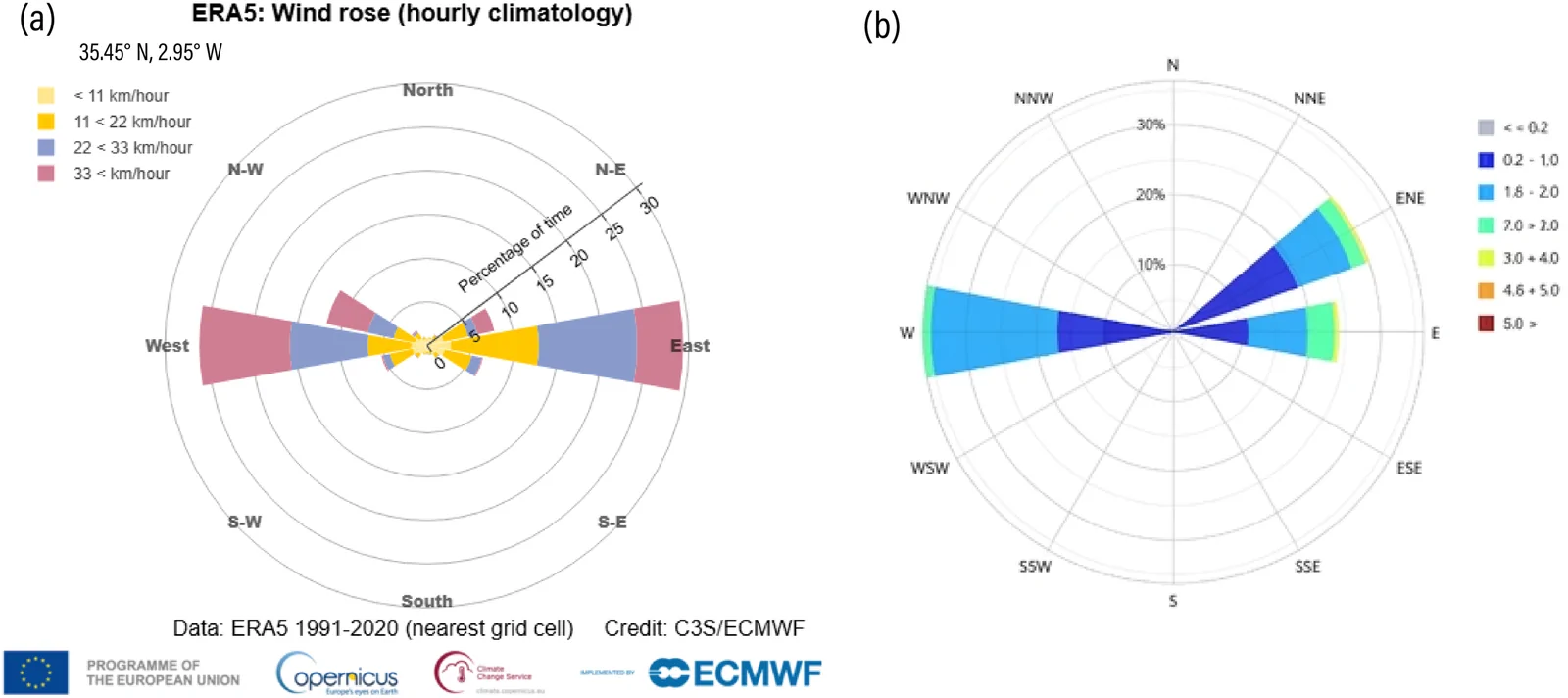
(a) Wind directions and speeds at 10 m altitude, (b) dominant wave rose at 36.00° N, 3.33° W.

The Almería–Oran Front, located along the eastern boundary of the study area, constitutes a major hydrodynamic discontinuity that governs water-mass separation and exerts a strong control on pollutant transport and dispersion processes [13]. Regional circulation is further modulated by the prevailing wind regimes, characterized by the alternation between Poniente (westerly) and Levante/Chergui (easterly) winds, which induce pronounced seasonal variability in hydrodynamic conditions [14]. The wave climate is predominantly influenced by northwestern (WNW) and northeastern (ENE) wave regimes (Fig 2). Overall, the study area is characterized by relatively moderate wave conditions, with mean significant wave heights averaging approximately 0.59 m and peak wave periods generally ranging between 6 and 9 s. Under normal conditions, significant wave heights commonly remain below 1.5 m [15]. Nevertheless, during winter, the Mediterranean basin is frequently affected by energetic storm events capable of generating significant wave heights exceeding 4 m and occasionally surpassing 5 m, thereby enhancing coastal hydrodynamics and sediment transport processes.

The selection of the simulation release point was based on the actual spatial distribution of vessels operating within the study area, as identified using real-time and historical ship traffic data available from the VesselFinder platform (https://www.vesselfinder.com/). The simulation parameters are summarized in Table 1. The selected scenario represents a major yet realistic oil pollution event, consistent with a plausible accident such as a fuel tank rupture or mechanical failure on a medium-sized tanker. This configuration enables an operational assessment of oil slick behaviour and the identification of coastal areas potentially exposed to hydrocarbon contamination along the NEMC.

**Table 1:** Simulation parameters and scientific justification.

Parameter	Configured Value	Scientific and Operational Justification
Release Area	North-eastern Moroccan Mediterranean coast	Shipping-exposed and ecologically sensitive corridor including Cap des Trois Fourches, Marchica Lagoon, Moulouya estuary, and Saïdia beaches
Release coordinates	Lat: 35.45° N, Lon: 2.95° W	Offshore Driouch/Nador sector; selected from vessel-activity exposure and proximity to vulnerable coastal systems
Start Date and Time	02 July 2024, 12:00 UTC	Summer high-impact scenario with strong stratification, sea-breeze influence, peak tourism, and high ecological vulnerability (see Section 2 for full justification)
Duration	168 hours (7 days)	Operationally relevant forecast horizon for early response and shoreline-impact assessment
Oil Type	Generic Bunker C / Heavy Fuel Oil)	Persistent commercial marine fuel; density = 971.1 kg m-3; initial kinematic viscosity approximately 2.17 x 10−2 m2 s-1
Released mass	100 metric tonnes	Significant spill requiring national and potentially transboundary response coordination
Particles	5,000	Provides stable trajectory statistics and mass-balance interpretation; each particle represents approximately 0.02 t
Initial release radius	1,000 m	Represents rapid early spreading before resolved advection, diffusion, and weathering dominate
Horizontal turbulent diffusivity	Kh = 10 m2 s-1	Represents unresolved subgrid dispersion through random-walk diffusion
Stokes Drift	Enabled	Required to represent wave-induced near surface transport and shoreward/alongshore drift
Vertical Mixing and weathering	Enabled	Represents entrainment, resurfacing, evaporation, emulsification, dispersion, biodegradation, and viscosity/density change

## 3. Materials and methods

### 3.1. OpenDrift/OpenOil framework

The numerical simulations performed in this study were conducted using OpenOil, a dedicated oil-spill module integrated into the open-source OpenDrift Lagrangian modeling framework (https://opendrift.github.io) developed by the Norwegian Meteorological Institute [9,16]. OpenDrift is a Python-based trajectory modeling system (version 1.14.2, Python 3.10), designed to simulate the transport and fate of drifting substances in marine environments using a Lagrangian particle-tracking approach governed by advection–diffusion processes [4]. Within this framework, oil particles are transported under the combined influence of ocean currents, wind forcing, wave-induced drift, and turbulent diffusion, allowing realistic representation of oil-spill dynamics in complex coastal environments.

**Fig 3:**
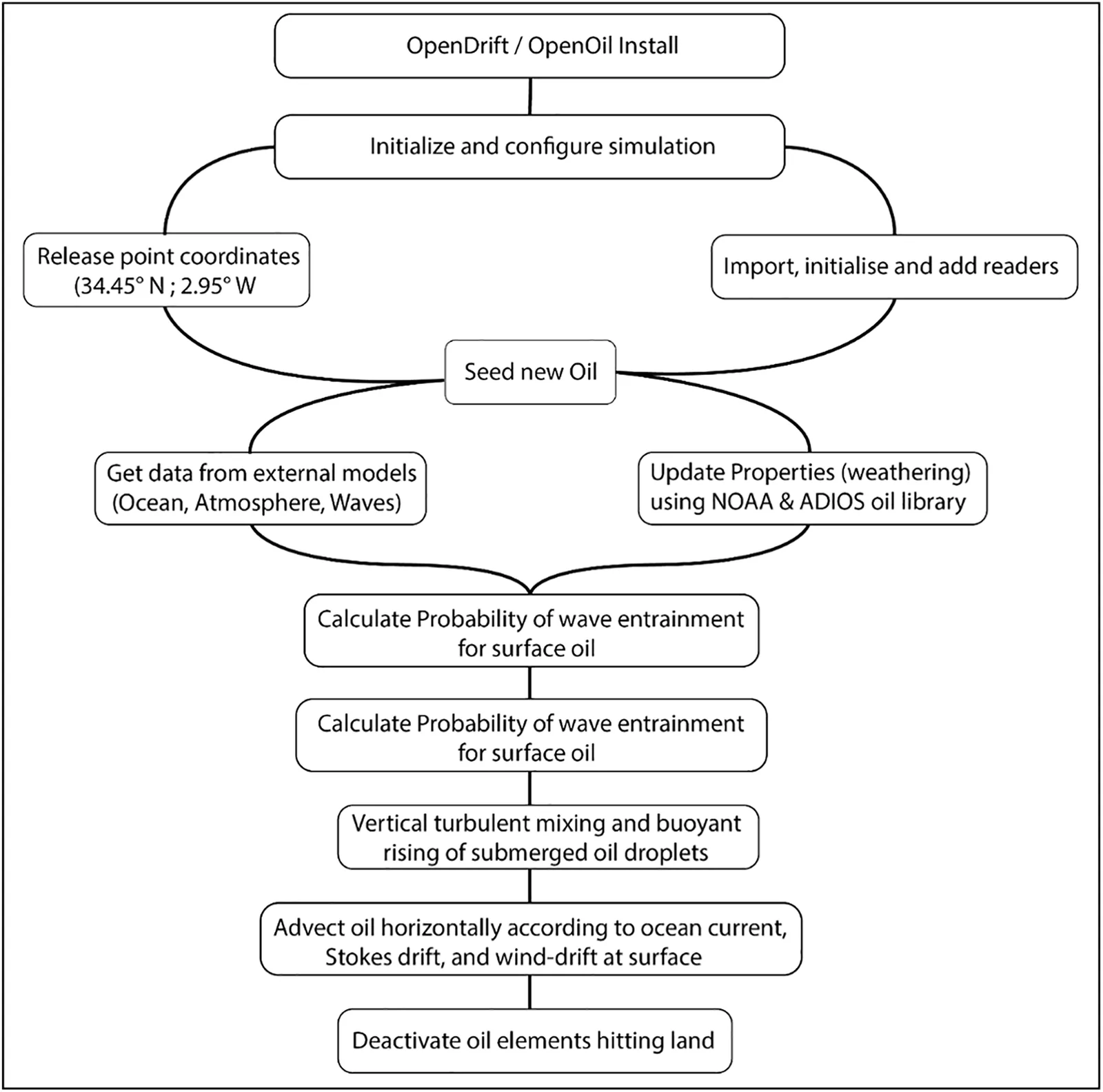
Flowchart illustrating the sequence of processes involved in OpenOil simulations.

The OpenOil module specifically simulates the transport and weathering of hydrocarbons by incorporating major physical and chemical transformation processes such as evaporation, entrainment, vertical mixing, resurfacing, emulsification, dispersion, and shoreline stranding. Oil-weathering parameterizations are derived from the NOAA ADIOS oil database, which provides detailed information on the physical and chemical properties of petroleum products under varying environmental conditions [17] (Fig 2). The overall methodological framework adopted in this study is presented in Fig 3.

### 3.2. Metocean forcing and model integration

To ensure realistic simulation of oil-spill transport and evolution, the OpenOil model was forced using operational meteorological and oceanographic datasets obtained from authoritative forecasting systems. Atmospheric forcing, including 10 m wind fields, was derived from the National Oceanic and Atmospheric Administration (NOAA) Global Forecast System (GFS), whereas oceanographic variables such as sea currents, sea level, temperature, salinity, and wave-related parameters were obtained from the Copernicus Marine Environment Monitoring Service (CMEMS). These operational products provide near-real-time environmental forecasts and are fully integrated within the OpenDrift framework, enabling continuous updates of boundary conditions throughout the simulation period [18].

The integration of high-resolution metocean forcing substantially improves the capability of the model to reproduce the complex hydrodynamic variability of the Alboran Sea, including mesoscale circulation structures, coastal currents, wind-driven transport, and wave-induced drift [19]. Such datasets are particularly important in semi-enclosed Mediterranean environments, where pollutant trajectories are highly sensitive to rapidly changing atmospheric and oceanographic conditions. The assimilation of realistic surface winds, ocean circulation fields, and wave parameters therefore enhances the reliability and physical consistency of oil-spill trajectory forecasts.

Within the OpenDrift framework, OpenOil simulates the transport and weathering of hydrocarbons through the interaction of several coupled physical processes, including advection, turbulent diffusion, evaporation, entrainment, vertical mixing, resurfacing, emulsification, and shoreline stranding. Vertical transport is controlled by the balance between wave entrainment, turbulent mixing, droplet buoyancy, and resurfacing processes. Breaking waves and upper-ocean turbulence may temporarily inject oil droplets into the water column, while buoyancy-driven rise velocity promotes their return toward the surface. Because the selected oil type (Generic Bunker C) is highly viscous, droplet breakup remains limited and natural dispersion is relatively weak compared with lighter refined oils, resulting in a strong persistence of oil near the sea surface despite episodic vertical excursions during energetic wind-wave conditions.

The oil-weathering parameterizations implemented in OpenOil are based on the NOAA ADIOS oil database, which provides detailed information on the physical and chemical properties of petroleum products under varying environmental conditions. In the present study, the selected oil type was Generic Bunker C (heavy fuel oil), characterized by a fresh-oil density of 971.1 kg m^−3^ and an initial kinematic viscosity of approximately 2.17 × 10^−2^ m² s^−1^. Although classified as a heavy oil, its fresh density remains lower than the average density of Mediterranean seawater, allowing the oil to initially remain afloat. However, progressive emulsification, sediment incorporation, and oil–mineral aggregate formation may subsequently increase the risk of partial sinking in shallow coastal environments.

For the numerical experiments, the spill scenario was configured as a release of 100 metric tonnes of Generic Bunker C distributed into 5,000 Lagrangian particles within an initial release radius of 1,000 m. The simulation started on 02 July 2024 at 12:00 UTC and was conducted over a period of 168 h (7 days). This scenario was designed to represent a major yet realistic accidental event, such as a fuel-tank rupture, bunker spill, or mechanical failure involving a medium-sized commercial vessel navigating through the Alboran Sea shipping corridor. Model outputs were analysed at hourly intervals to evaluate particle trajectories, shoreline stranding, oil mass balance, weathering evolution, and the influence of hydrodynamic forcing along the main transport pathways.

Recent developments in the OpenDrift/OpenOil framework have further enhanced forecasting performance through the integration of data-driven correction approaches, including Adversarial Temporal Convolutional Networks (ATCNs), which improve the representation of sea-surface dynamics and strengthen agreement with ocean reanalysis products [20]. These advances contribute to more accurate prediction of oil-spill trajectories and improve the operational applicability of numerical drift simulations in coastal risk management.

## 4. Results and discussion

### 4.1. Hydrodynamic forcing during the simulated event

The revised analysis explicitly links slick evolution to the met-ocean forcing extracted along the release point and the main particle cloud. During the first 72 h, 10 m winds were weak to moderate, ranging approximately from 2.1 to 7.8 m s^−1^, with dominant W-WNW components during the early phase and more variable easterly/north-easterly components later in the simulation. These directional changes are consistent with the observed slick elongation followed by partial recirculation near the coast.

Wave conditions were moderate for the summer season. Significant wave height ranged approximately from 0.35 to 1.45 m, and mean/peak wave periods were generally between 3.8 and 6.9 s. Wave directions broadly followed the wind regime, producing Stokes-drift components that enhanced near-surface alongshore and shoreward transport during wave events. Surface current speeds varied approximately from 0.04 to 0.32 m s^−1^, with the strongest east-southeastward components associated with the North African margin circulation and mesoscale structures of the eastern Alboran Sea.

**Fig 4:**
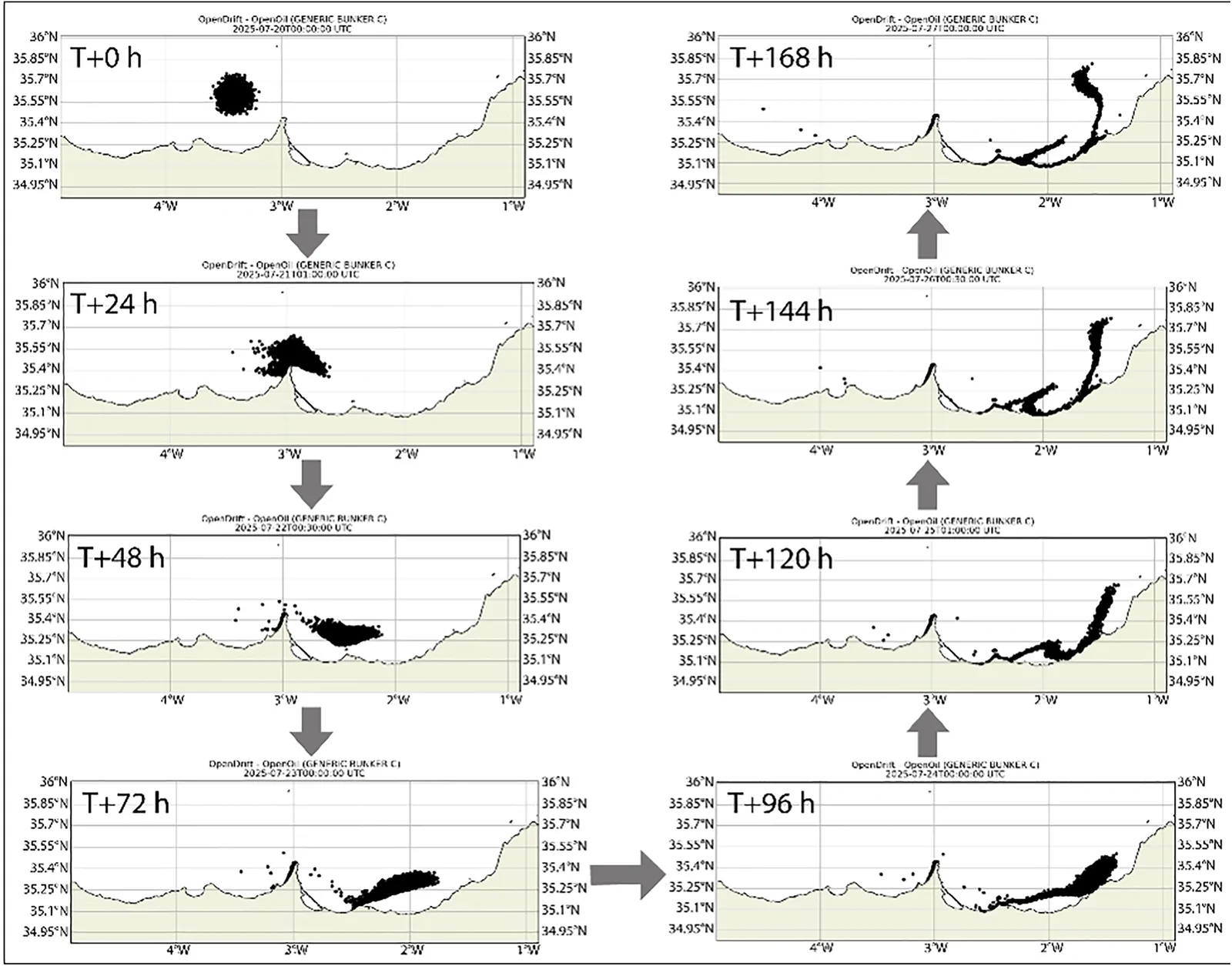
Spatio-temporal evolution of the simulated oil slick (Generic Bunker C) along the North-eastern Moroccan Mediterranean coast from 20 to 27 July 2025.

These values show that the spill was not driven by a single forcing term. Instead, the early trajectory reflects the combined action of eastward coastal currents, direct wind drift on floating oil, and Stokes drift. Later recirculation and renewed coastal accumulation reflect the sensitivity of nearshore pathways to changes in wind direction and mesoscale current variability. Time series of wind, wave, and current forcing are displayed in Fig 4.

### 4.2. Horizontal transport, dispersion, and shoreline stranding

The OpenDrift–OpenOil simulation enables a detailed reconstruction of the temporal evolution and spatial fate of a hypothetical oil spill of 100 metric tonnes of bunker fuel oil released in the eastern Alboran Sea. The spill is discretized into 5,000 Lagrangian particles, each representing 0.02 metric tonnes, allowing a quantitative interpretation of oil mass distribution, transport pathways, and coastal contamination intensity.

At the onset of the release (T + 0 h), the oil slick remains compact and localized near the release point. During the first 24 hours (T + 24 h), the slick undergoes rapid elongation along a northwest–southeast axis, driven by the combined influence of westerly winds, wind drift on the floating fraction, wave-induced Stokes drift, and the eastward coastal current component along the North African margin. This phase marks the transition from a localized spill to a dynamically advected slick. Between T + 48 h and T + 72 h, the slick exhibits progressive stretching and partial bifurcation. A fraction of the particles is transported offshore under the influence of mesoscale structures linked to the eastern Alboran Gyre, while the main body remains in the nearshore corridor. The simulated pattern indicates that offshore dilution capacity is limited under the selected summer conditions and that small changes in wind and wave direction can redirect oil toward sensitive shoreline segments.

**Fig 5:**
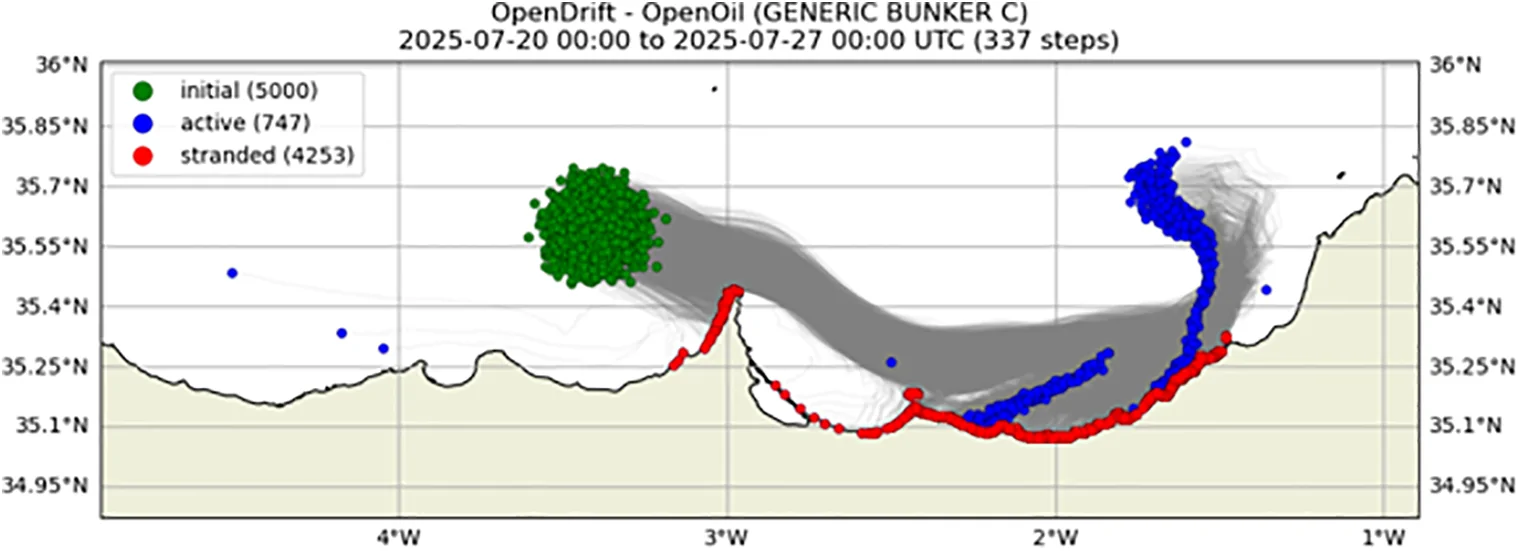
Single time-of-arrival map summarising the dispersion process, with colour ramp encoding the day of shoreline stranding from Day 1 to Day 7.

From T + 96 h to T + 120 h, the simulation indicates that part of the oil mass reaches the western Algerian coastline, particularly in the Ghazaouet–Beni Saf sector, highlighting the strong hydrodynamic connectivity between the northeastern Moroccan and western Algerian coasts and confirming the transboundary nature of oil spill risks. During the final phase (T + 144 h to T + 168 h), partial westward re-advection leads to renewed exposure of the Saïdia–Nador sector.

approximately 85.1 tonnes of oil, or more than 80% of the total released mass (Fig 5). This high stranding rate indicates a limited offshore dispersion capacity and underscores the strong coastal retention characteristic of the NEMC region. The residual floating fraction (747 particles, ~ 14.9 t) remains operationally important because it can fragment, re-strand, or move across national boundaries under subsequent changes in winds and currents.

### 4.3. Oil budget, weathering, density, and viscosity evolution

**Fig 6:**
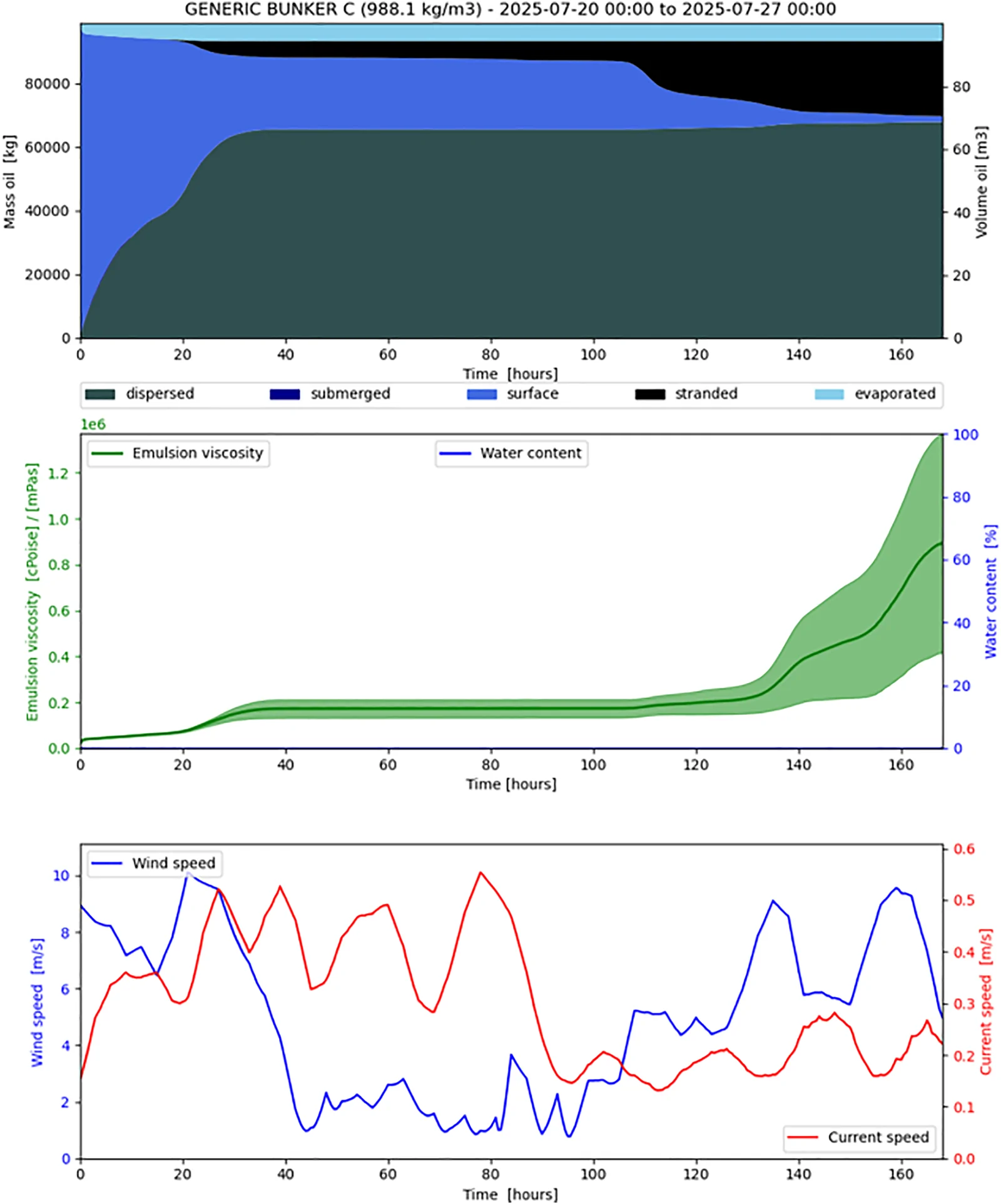
Temporal evolution of oil weathering, mass balance, emulsion properties, and hydrodynamic forcing during the simulation period.

The oil budget diagram provides a comprehensive overview of the physical and chemical fate of the spilled hydrocarbon over the seven-day simulation period (Fig 6). For the Generic Bunker C fuel, the temporal evolution of the different mass compartments clearly highlights the highly persistent nature of this heavy fuel oil.

The oil-budget results confirm the persistent character of Generic Bunker C. Evaporation is rapid during the first hours but remains limited, reaching approximately 10–15% of the released mass after 24–48 h. This limited loss is expected for residual heavy fuel oils dominated by high-molecular-weight fractions and relatively low volatile content [21,22]. Natural dispersion remains below 10% throughout the simulation. The surface oil fraction decreases only gradually, and after 168 hours more than 60% of the initially released mass remains either floating or stranded along the coastline.

The revised analysis quantifies oil-property changes more explicitly. The fresh-oil density of 971.1 kg m^−3^ increases during weathering and emulsification, reaching values approaching approximately 990–1005 kg m^−3^ in the later stages of the simulation. The dynamic viscosity of the oil-water emulsion increases from approximately 2.0 × 10⁴ cP to values on the order of 1.0 × 10^5^ to 3.0 × 10^5^ cP, depending on water content and temperature — a five- to fifteenfold increase. Water content rises rapidly and reaches approximately 70–80% within the first 48 h, producing a three- to fourfold increase in the volume of material requiring storage and handling.

This behaviour explains the apparent paradox between heavy-oil classification and surface residence. Bunker C is heavy and viscous relative to light refined products, but the fresh configured density remains below seawater density (~1027–1029 kg m^−3^), so it initially floats. Sedimentation is therefore not dominant immediately after release. Instead, sinking risk becomes important in shallow, sediment-rich areas where weathered oil can incorporate suspended mineral particles, form oil-mineral aggregates, approach or exceed ambient seawater density, and create persistent subsurface tarballs or benthic contamination [22,23]. This mechanism represents a critical concern near the Moulouya estuary.

On the simulated time scale, biodegradation plays a negligible role, contributing to less than 5% of mass loss. This confirms that biological processes do not provide an effective short-term mitigation pathway for heavy fuel oil spills and reinforces the need for active response measures rather than reliance on natural attenuation [24].

The evolution of oil properties further emphasizes the operational challenges associated with Bunker C spills. Such viscosities render conventional weir skimmers ineffective and significantly reduce the efficiency window for chemical dispersants, which typically lose effectiveness once viscosity exceeds 2000–5000 cP. Consequently, recovery operations would require specialized mechanical equipment, such as oleophilic brushes or screw pumps.

### 4.4. Vertical transport, wave entrainment, resurfacing, wind drift, and Stokes drift

The vertical distribution of particles indicates that most oil remains in the upper surface layer, but episodic wind-wave events can entrain a small fraction of droplets to depths of approximately 5–10 m. Three competing processes control this behaviour. First, wave breaking and turbulent mixing inject oil droplets downward, especially during short periods of elevated wind speed and wave height. Second, buoyancy-driven resurfacing returns droplets upward, with the rise velocity depending on droplet size, density contrast, and viscosity. Third, stratification and vertical shear can separate surface and subsurface pathways, causing temporary deviations between visible slick movement and subsurface droplet motion.

For the selected Bunker C oil, resurfacing and surface retention dominate over sustained subsurface dispersion. High viscosity inhibits the production of small droplets, so entrainment is intermittent and does not create a large, persistent dispersed fraction. Nevertheless, the temporary vertical excursions are environmentally relevant because they increase the oil-water interfacial area and expose plankton, fish larvae, and nearshore pelagic organisms to contamination during the early phase of the spill.

Wind drift and Stokes drift exert complementary effects on the horizontal dispersion. Direct wind drift controls the early downwind elongation of the floating slick and accelerates shoreline approach when winds have an onshore component. Stokes drift, generated by the surface wave field, adds a wave-coherent transport component that is strongest near the surface and decays with depth. Under the moderate summer wave conditions simulated here (Hs up to 1.45 m), Stokes drift contributes to alongshore and shoreward displacement, increasing the likelihood that oil remains trapped in the nearshore corridor rather than being exported offshore. The resolved ocean currents provide the background east-southeastward transport and transboundary connectivity. A complementary sensitivity analysis (with and without Stokes drift) is identified in Section 5 as a recommended follow-up.

### 4.5. Environmental vulnerability and socio-ecological implications

The Marchica Lagoon emerges as a critical hotspot of environmental vulnerability within the study area. The model identifies a nearshore exposure pathway capable of transporting oil contaminated waters toward the Boucana inlet. However, the present 1/24° hydrodynamic forcing cannot resolve the detailed morphology, tidal jets, inlet exchange, or operational closure dynamics of the inlet. Therefore, the results should be interpreted as evidence of potential exposure at the lagoon entrance, not as a fully resolved prediction of internal lagoon circulation. Any emergency closure, boom deployment, or inlet-protection operation must be planned with local high-resolution hydrodynamic modelling, tidal information, and environmental feasibility assessment.

If oil enters the lagoon, the risk of long residence time and chronic contamination is high. Under such conditions, stranded and floating oil would preferentially accumulate on intertidal mudflats and within seagrass meadows dominated by Cymodocea nodosa and Zostera noltii. These habitats are of high ecological importance, providing nursery and spawning grounds for fish species and feeding areas for migratory birds, including flamingos and spoonbills [25]. The deposition and persistence of PAHs within lagoonal sediments would result in chronic contamination. Existing anthropogenic pressures on the Marchica Lagoon further reduce ecosystem resilience [24,26].

The rocky coastline of Cap des Trois Fourches is likewise identified as highly exposed. The giant limpet (Patella ferruginea), an endemic species listed as critically endangered, inhabits the intertidal rocky zone and would be directly affected by oil smothering. Seabirds nesting along the cliffs, including Audouin's gull (Larus audouinii) and the Cory's shearwater, face a high risk of lethal oiling through plumage contamination [27]. Moreover, the steep and inaccessible nature of this coastline would severely constrain clean-up operations [28].

From a socio-economic perspective, the eastern Moroccan coast is highly dependent on seasonal beach tourism, with activity strongly concentrated in Saïdia during the summer months. The July-based simulation indicates that oil contamination could reach the beaches of Saïdia within less than one week. Artisanal fisheries between Nador and Saïdia could be affected by immediate fishery closures, tainting of seafood products, loss of consumer confidence, and longer-term concerns about PAH bioaccumulation. Because the simulation indicates possible connectivity toward western Algeria, response planning should also include transboundary notification and coordination mechanisms.

The dynamics observed in this study are consistent with previous modelling efforts in the Mediterranean Sea. Simulations conducted in the Saronic Gulf (Greece) using OpenOil have demonstrated a comparable sensitivity of semi-enclosed coastal systems to pollutant accumulation [29]. Similarly, analyses of historical Mediterranean oil spills highlighted the dominant role of coastal currents and Stokes drift in driving shoreline contamination [30]. In contrast with modelling studies along the Moroccan Atlantic coast [4], dispersion in the NEMC region appears more strongly constrained by coastal geometry and less governed by linear oceanic drift, resulting in enhanced coastal retention and prolonged environmental exposure.

## 5. Conclusion

This study provides a process-based numerical assessment of heavy fuel oil dispersion and weathering along the north-eastern Moroccan Mediterranean coast using the OpenOil module of OpenDrift — the first such operationally oriented simulation for the Nador–Saïdia sector. The main scientific finding is that a 100 t Generic Bunker C spill released offshore Driouch during the selected summer conditions can strand rapidly along the NEMC, with more than 80% of the released mass reaching the shoreline within one week. The transport pathway is controlled by the combined influence of Alboran mesoscale circulation, the Algerian Current, local wind drift, Stokes drift, wave entrainment, and nearshore retention.

The weathering results show that evaporation and natural dispersion are secondary for this heavy fuel oil, whereas emulsification, water uptake, viscosity increase, and surface persistence dominate the early fate of the spill. The fresh oil floats because its density is lower than seawater, but sedimentation risk becomes important after weathering and in shallow, sediment rich environments where oil-mineral aggregates can form. This distinction resolves the apparent contradiction between heavy-oil classification, long surface residence, and potential benthic contamination.

From a management perspective, the results support three preparedness priorities: (i) prepositioning response equipment suitable for highly viscous and emulsified oils; (ii) preparing protection strategies for high-value coastal receptors, including Marchica Lagoon, the Moulouya estuary, Cap des Trois Fourches, and Saïdia beaches; and (iii) integrating numerical forecasts, satellite observations, and transboundary coordination into national spill-response planning. Protection of the Boucana inlet should be considered as an operational option only where hydrodynamic, environmental, and logistical assessments confirm feasibility.

The study also has limitations. The model resolution does not resolve narrow inlet dynamics, small harbour-scale currents, or detailed shoreline clean-up accessibility. The analysis is based on one summer scenario and should be complemented by winter-storm, multi-year ensemble, and sensitivity simulations with and without wind drift, Stokes drift, and alternative oil types. Future work should couple OpenDrift with higher-resolution coastal hydrodynamics, SAR based slick detection, real-time vessel traffic, and probabilistic risk mapping to strengthen operational early-warning capacity for Morocco's Mediterranean coast.

## Supporting information

S1 File Contains the raw data. (ZIP)

S2 File Contains additional and detailed data supporting the study. (ZIP)

## References

[1] Fingas M. Introduction to Oil Spills and their Clean-up. Petrodiesel Fuels. CRC Press; 2021. p. 875–89. doi: 10.1201/9780367456252-4

[2] Polinov S, Bookman R, Levin N. Spatial and temporal assessment of oil spills in the Mediterranean Sea. Mar Pollut Bull. 2021;167:112338. doi: 10.1016/j.marpolbul.2021.112338 33940431

[3] Alves TM, Kokinou E, Zodiatis G, Radhakrishnan H, Panagiotakis C, Lardner R. Multidisciplinary oil spill modeling to protect coastal communities and the environment of the Eastern Mediterranean Sea. Sci Rep. 2016;6:36882. doi: 10.1038/srep36882 27830742 PMC5103274

[4] Haddaoui H, Hakkou M, Aangri A, Souiri S, Amari YS, Laghzal A. Numerical simulation of oil spill dispersion along Morocco’s northern Atlantic coast: A case study near Asilah. Ecol Eng Environ Technol. 2025;26: 310–22. doi: 10.12912/27197050/203450

[5] Câmara SF, Pinto FR, Silva FR, Soares MO, De Paula TM. Socioeconomic vulnerability of communities on the Brazilian coast to the largest oil spill (2019–2020) in tropical oceans. Ocean & Coastal Management. 2021;202:105506. doi: 10.1016/j.ocecoaman.2020.105506

[6] Souiri S, Hakkou M, Aangri A, Belattmania A, Castelle B. Multidecadal satellite-derived sandy shoreline change along the Mediterranean coast of Morocco. Regional Studies in Marine Science. 2026;93:104667. doi: 10.1016/j.rsma.2025.104667

[7] Ouagga T, Sahib N. Evaluating management effectiveness of the Moulouya River Estuary Ramsar Site in Morocco: an application of the R-METT tool and implications for conservation. Marine and Freshwater Research. 2025;76(5). doi: 10.1071/mf24264

[8] Oubdil S, Souiri S, Ajmani S, Nazih A, Mentag R, Benradi F, et al. Characterization and GIS Mapping of the Physicochemical Quality of Soils in the Irrigated Area of Tafrata (Eastern Morocco): Implications for Sustainable Agricultural Management. Geographies. 2025;5(4):66. doi: 10.3390/geographies5040066

[9] Dagestad K-F, Röhrs J, Breivik Ø, Ådlandsvik B. OpenDrift v1.0: a generic framework for trajectory modelling. Geosci Model Dev. 2018;11(4):1405–20. doi: 10.5194/gmd-11-1405-2018

[10] Vennell R, Scheel M, Weppe S, Knight B, Smeaton M. Fast lagrangian particle tracking in unstructured ocean model grids. Ocean Dynamics. 2021;71(4):423–37. doi: 10.1007/s10236-020-01436-7

[11] Souiri S, Hakkou M, Haddaoui H, Oubdil S, Lmrimed N. Automatic Coastline Detection Using Coastsat Toolbox and GIS: A Case Study at Oued-Laou Beach, Morocco. E3S Web Conf. 2025;607:04024. doi: 10.1051/e3sconf/202560704024

[12] Aguiar E, Mourre B, Juza M, Reyes E, Hernández-Lasheras J, Cutolo E, et al. Multi-platform model assessment in the Western Mediterranean Sea: impact of downscaling on the surface circulation and mesoscale activity. Ocean Dynamics. 2019;70(2):273–88. doi: 10.1007/s10236-019-01317-8

[13] Sanchez-Vidal A, Calafat A, Canals M, Fabres J. Particle fluxes in the Almeria-Oran Front: control by coastal upwelling and sea surface circulation. Journal of Marine Systems. 2004;52(1–4):89–106. doi: 10.1016/j.jmarsys.2004.01.010

[14] Souiri S, Hakkou M, Haddaoui H, Aangri A, Oubdil S, Chtioui T. Monthly analysis of oceanographic conditions in the western mediterranean sea. Integration of Advanced Systems in Environmental Science and Water Desalination. CRC Press; 2025. p. 189–94. doi: 10.1201/9781003647263-17

[15] El Mrini A, Anthony EJ, Taaouati M, Nachite D, Maanan M. A note on contrasting morphodynamics of two beach systems with different backshores, Tetouan coast, northwest Morocco: the role of grain size and human-altered dune morphology. Journal of Coastal Research. 2013;165:1283–8. doi: 10.2112/si65-217.1

[16] Dagestad K-F, Rohrs J, Idzanovic M, Rikardsen E, Hope G. Using Ensemble Ocean Currents for Drift Predictions. 2025. [cited 25 May 2026]. doi: 10.5194/egusphere-egu24-15769

[17] Röhrs J, Dagestad K-F, Asbjørnsen H, Nordam T, Skancke J, Jones CE, et al. The effect of vertical mixing on the horizontal drift of oil spills. Ocean Sci. 2018;14(6):1581–601. doi: 10.5194/os-14-1581-2018

[18] Keramea P, Kokkos N, Gikas G, Sylaios G. Operational Modeling of North Aegean Oil Spills Forced by Real-Time Met-Ocean Forecasts. JMSE. 2022;10: 411. doi: 10.3390/jmse10030411

[19] Prasad SJ, Francis PA, Balakrishnan Nair TM, Shenoi SSC, Vijayalakshmi T. Oil spill trajectory prediction with high-resolution ocean currents. Journal of Operational Oceanography. 2019;13(2):84–99. doi: 10.1080/1755876x.2019.1606691

[20] Ren P, Jia Q, Xu Q, Li Y, Bi F, Xu J, et al. Oil spill drift prediction enhanced by correcting numerically forecasted sea surface dynamic fields with adversarial temporal convolutional networks. IEEE Trans Geosci Remote Sensing. 2025;63:1–18. doi: 10.1109/tgrs.2025.3528631

[21] Boehm PD, Page DS, Brown JS, Neff JM, Bragg JR, Atlas RM. Distribution and weathering of crude oil residues on shorelines 18 years after the Exxon Valdez spill. Environ Sci Technol. 2008;42(24):9210–6. doi: 10.1021/es8022623 19174894

[22] Levy DA. Pipelines and salmon in northern British Columbia. Pembina Institute Drayton Valley; 2009.

[23] Radović JR, Domínguez C, Laffont K, Díez S, Readman JW, Albaigés J, et al. Compositional properties characterizing commonly transported oils and controlling their fate in the marine environment. J Environ Monit. 2012;14(12):3220–9. doi: 10.1039/c2em30385j 23117332

[24] Zerrouqi Z, Sbaa M, Chafi A, Aqil H. Contribution à l’étude de la qualité des eaux de la lagune de Nador: Impact de l’anthropisation. Bulletin de l’Institut Scientifique, Rabat, Section Sciences de la Vie. 2013;35: 51–9.

[25] Papaioannou V, Anagnostopoulos CGE, Vlachos K, Moumtzidou A, Gialampoukidis I, Vrochidis S, et al. Modeling seabed-origin oil spills using OpenOil: Case studies from the Mediterranean Sea. OJMS. 2025;15: 129–56. doi: 10.4236/ojms.2025.153008

[26] Hlal M, El Monhim B, Bouymajjan M, Sbai A, Benrbia K, Cwick G, et al. Impact of human activities on the Marchica lagoon ecosystem in northeast Morocco. E3S Web of Conferences. EDP Sciences; 2025. p. 04014.

[27] Espinosa F, Navarro-Barranco C, González AR, Maestre M, García-Gómez JC, Benhoussa A, et al. A combined approach to assessing the conservation status of Cap des Trois Fourches as a potential MPA: is there a shortage of MPAs in the Southern Mediterranean? Mediterranean Marine Science. 2014; 654–66.

[28] Weatherall P, Ferreras SC, Cardigos SD, Cornish N, Davidson SR, Dorschel B, et al. The GEBCO_2024 Grid-a continuous terrain model of the global oceans and land. 2024.

[29] Papaioannou V, Anagnostopoulos CGE, Vlachos K, Moumtzidou A, Gialampoukidis I, Vrochidis S, et al. OpenOil-Based Analysis of Oil Dispersion Dynamics: The Agia Zoni II Shipwreck Case. Water. 2025;17(14):2126. doi: 10.3390/w17142126

[30] Liubartseva S, Coppini G, Daniel P, Melaku Canu D, Hoxhaj M. Model-based insights into pathways and fate of oil spills in the Mediterranean Sea. Mar Pollut Bull. 2025;217:118061. doi: 10.1016/j.marpolbul.2025.118061 40349612

